# Supplementary material for: Assessing the Impact of External and Internal Factors on Emergency Department Overcrowding
Source: Healthcare (Basel). 2025 Oct 14;13(20):2577. doi: 10.3390/healthcare13202577 (PMC12562336; doi:10.3390/healthcare13202577)
Supplement: Supplementary file 1 [file healthcare-13-02577-s001.zip › healthcare-3834098-supplementary.docx]

# Supplementary Materials

Table S1. Model design.

|  | **Baseline Model** | **Model 2: 3 Hours Before** | **Model 3: 6 Hours Before** | **Model 4: 12 Hours After** | **Model 5: 24 Hours After** | **Model 6: Rolling Averages** | **Model 7: Rolling Averages 2** |
| --- | --- | --- | --- | --- | --- | --- | --- |
| Football Game Actual Time | x |  |  |  |  | x | x |
| Hospital Census | x |  |  | x | x |  |  |
| Boarding Count | x |  |  | x | x |  |  |
| Federal Holiday | x | x | x | x | x | x | x |
| Weather Clear | x |  |  | x | x | x | x |
| Weather Clouds | x |  |  | x | x | x | x |
| Weather Rain | x |  |  | x | x | x | x |
| Weather Thunderstorm | x |  |  | x | x | x | x |
| Weather Others | x |  |  | x | x | x | x |
| Month | x | x | x | x | x | x | x |
| Day Of Month | x | x | x | x | x | x | x |
| Hour | x | x | x | x | x | x | x |
| Day Of Week | x | x | x | x | x | x | x |
| Heat Index | x | x | x | x | x | x | x |
| Wind Speed | x |  |  | x | x | x | x |
| Weather Clear 3 Hours Before |  | x |  |  |  |  |  |
| Weather Clouds 3 Hours Before |  | x |  |  |  |  |  |
| Weather Rain 3 Hours Before |  | x |  |  |  |  |  |
| Weather Thunderstorm 3 Hours Before |  | x |  |  |  |  |  |
| Weather Others 3 Hours Before |  | x |  |  |  |  |  |
| Wind Speed 3 Hours Before |  | x |  |  |  |  |  |
| Treatment Count 3 Hours Before |  | x |  |  |  |  |  |
| Boarding Count 3 Hours Before |  | x |  |  |  |  |  |
| Hospital Census 3 Hours Before |  | x |  |  |  |  |  |
| Football Game 12 Hours Before |  | x |  |  |  |  |  |
| Football Game 12 Hours After |  |  |  | x |  |  |  |
| Weather Clear 6 Hours Before |  |  | x |  |  |  |  |
| Weather Clouds 6 Hours Before |  |  | x |  |  |  |  |
| Weather Rain 6 Hours Before |  |  | x |  |  |  |  |
| Weather Thunderstorm 6 Hours Before |  |  | x |  |  |  |  |
| Weather Others 6 Hours Before |  |  | x |  |  |  |  |
| Wind Speed 6 Hours Before |  |  | x |  |  |  |  |
| Treatment Count 6 Hours Before |  |  | x |  |  |  |  |
| Boarding Count 6 Hours Before |  |  | x |  |  |  |  |
| Hospital Census 6 Hours Before |  |  | x |  |  |  |  |
| Football Game 24 Hours Before |  |  | x |  |  |  |  |
| Football Game 24 Hours After |  |  |  |  | x |  |  |
| Rolling Mean Treatment Count Window Size 6 |  |  |  |  |  | x | x |
| Rolling Mean Boarding Count Window Size 6 |  |  |  |  |  | x | x |
| Rolling Mean Hospital Census Window Size 12 |  |  |  |  |  | x |  |
| Rolling Mean Hospital Census Window Size 24 |  |  |  |  |  |  | x |


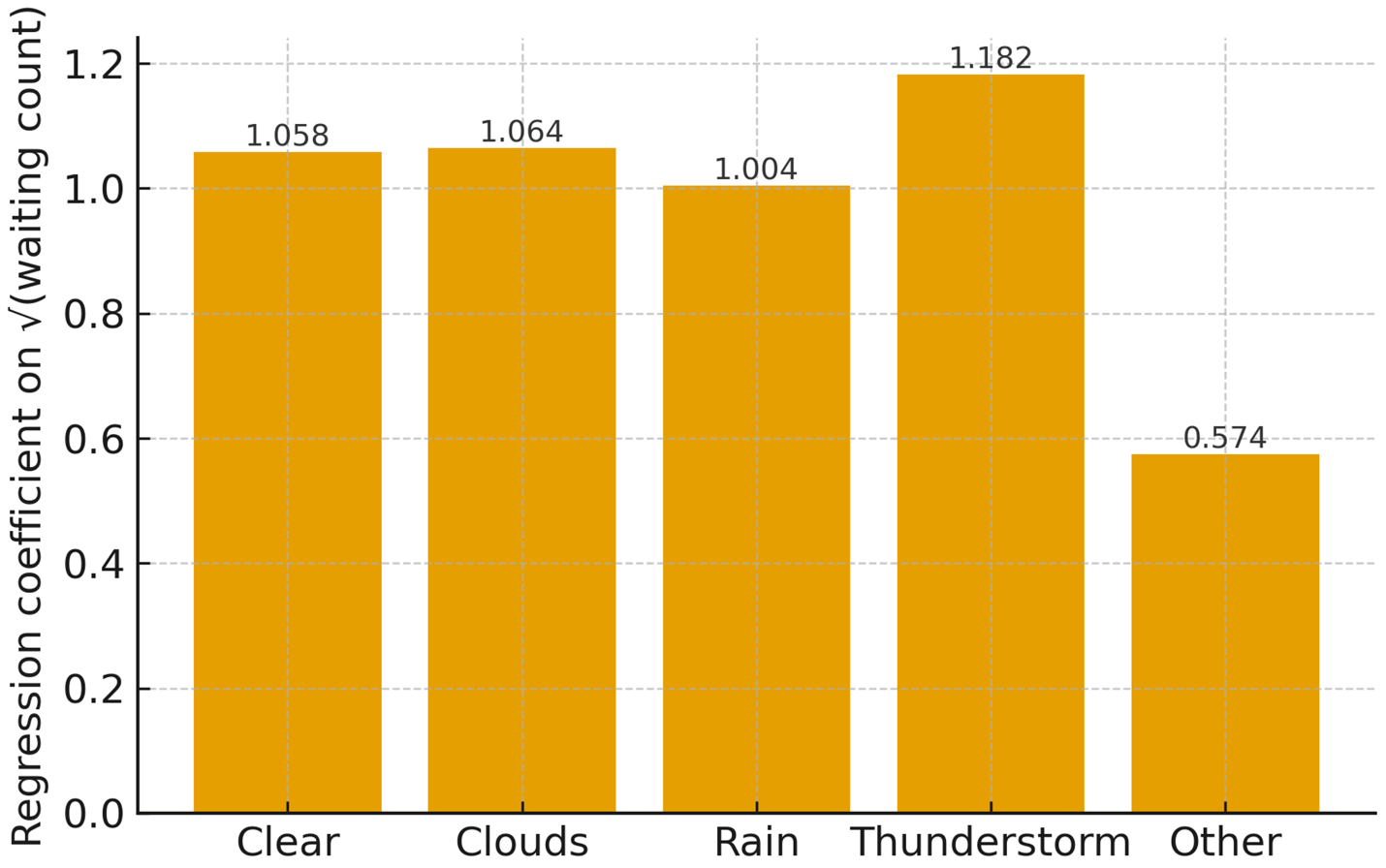


Figure S1. Weather effects (Baseline Model).


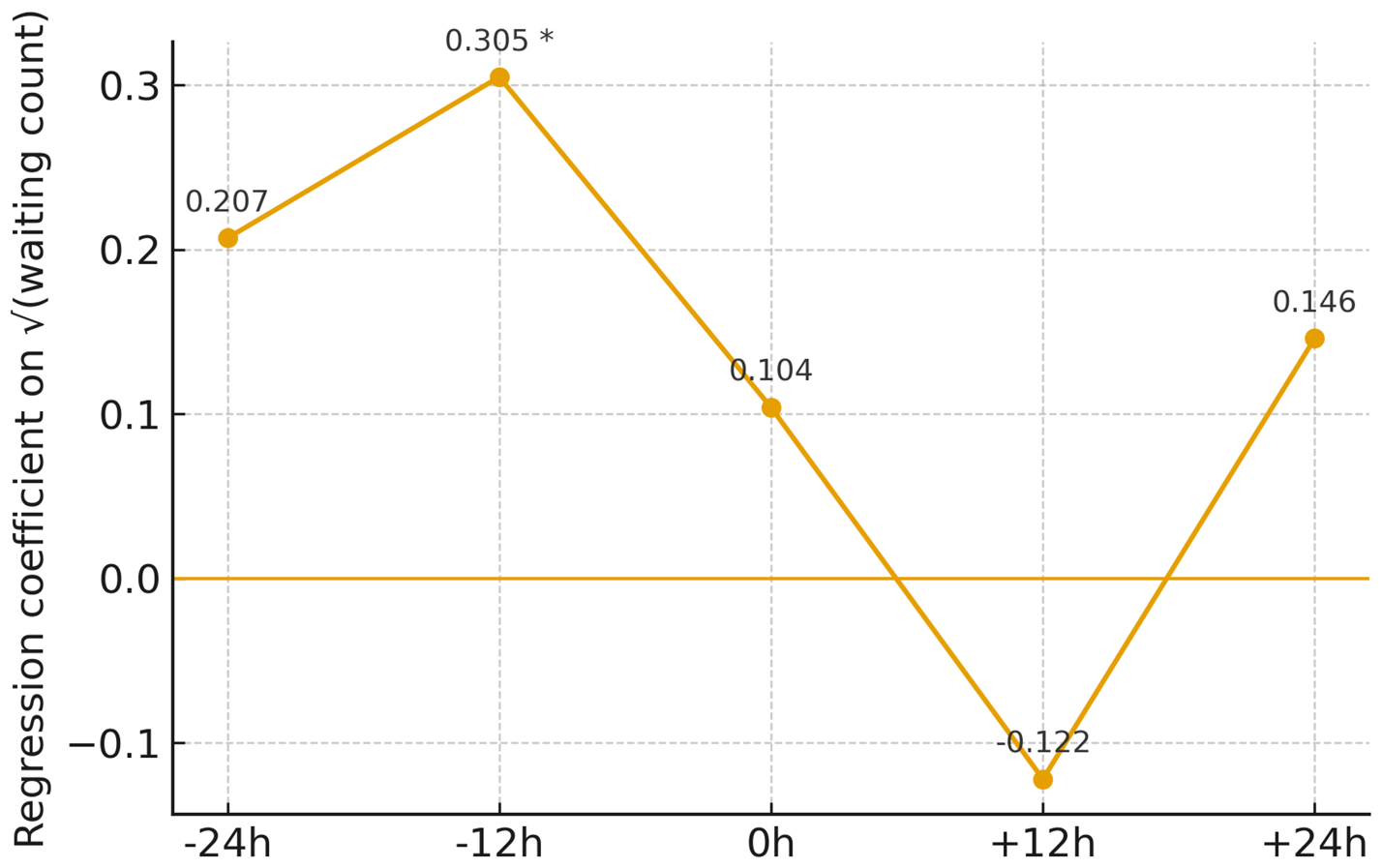


Figure S2. Football game timing effects (event time).


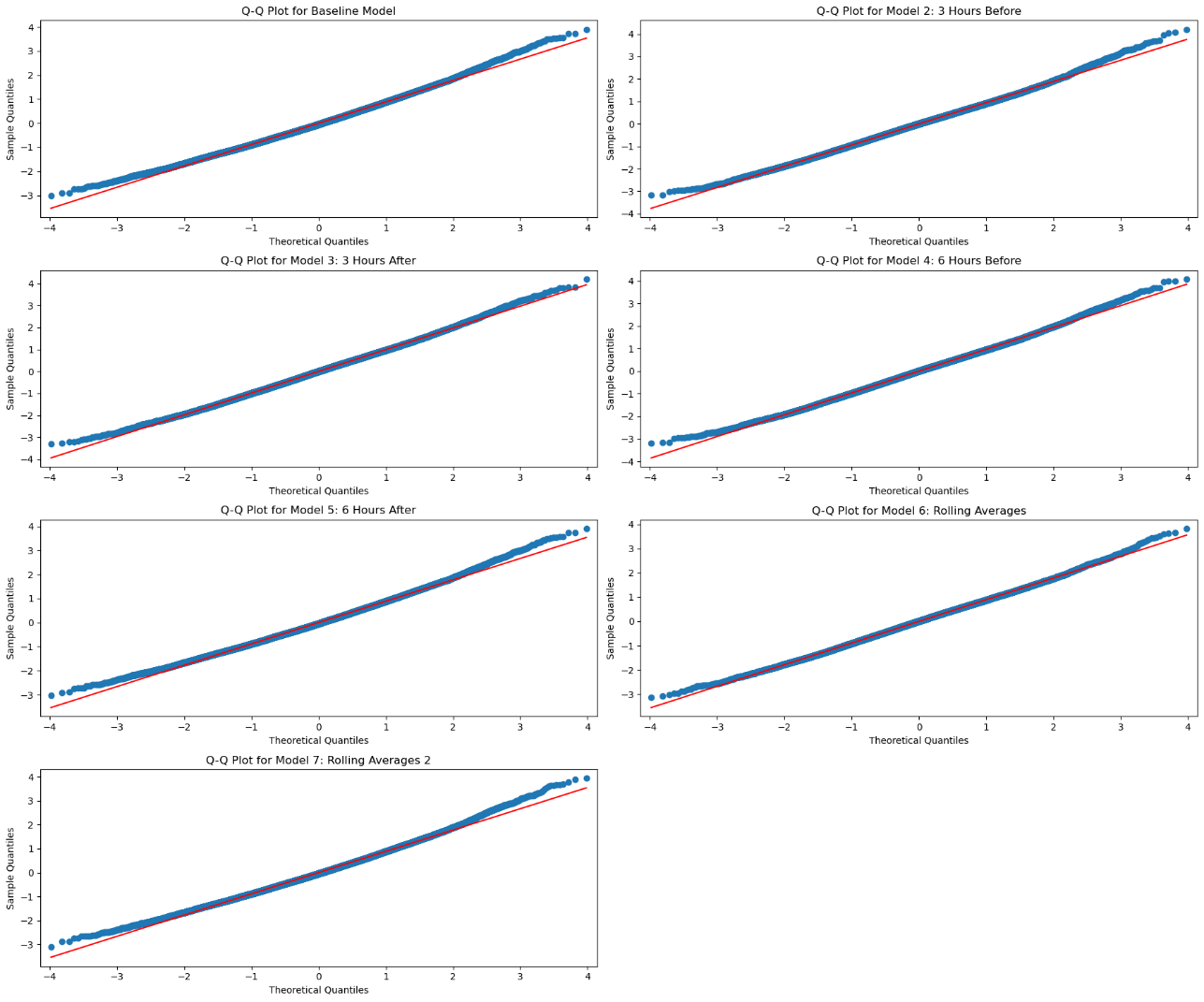


Figure S3. Normality test for the residuals of all models.
